# Nucleoside Modifications and Poly(A) Tail Length Greatly Influence Protein Expression from In Vitro-Transcribed mRNA in a Salmonid Cell Line

**DOI:** 10.3390/vaccines14050367

**Published:** 2026-04-22

**Authors:** Thea Fossum Krog, Ida Soo Haukland, Gyri Teien Haugland

**Affiliations:** Department of Biological Sciences, University of Bergen, 5006 Bergen, Norway; thea.krog@uib.no (T.F.K.);

**Keywords:** mRNA vaccine, modified nucleosides, poly(A) tail, salmon, in vitro transcription

## Abstract

Background/Objectives: It is challenging to develop efficient vaccines against intracellular pathogens such as viruses, and since viral infections are one of the main challenges for farmed salmon, a novel vaccine strategy is needed. mRNA vaccines are optimized and approved for humans, but for fish, the mRNA technology is new, and optimization is required to ensure efficient protein expression. We made an mRNA tailored to salmon and studied the effect of modified nucleosides and the length of the poly(A) tail on protein expression from in vitro-transcribed mRNA in CHSE-214 cells, using enhanced green fluorescent protein (EGFP) as a reporter. Methods: Different lengths of the poly(A) tail were tested, and various modified nucleotides were incorporated in the mRNA during in vitro transcription, including pseudouridine (Ψ), N1-methylpseudouridine (m1Ψ), N6-methyladenosine (m6A), 5-methyluridine (m5U), and 5-methylcytidine (m5C). Protein expression was observed in fluorescence microscopy and quantified using flow cytometry. Results: mRNA containing Ψ resulted in the strongest EGFP expression 1–3 days post-transfection (dpt), while EGFP expression from m5C mRNA was high throughout the experiment (<10 dpt). m5U-containing mRNA had low EGFP expression until 6 dpt, but reached the level of m5C mRNA at 10 dpt. The m5U mRNA, however, expressed EGFP at much higher intensity than all the other mRNAs at all time points. Poly(A) tails with lengths of 40, 100, and >100 were tested, and the one with >100 adenines showed the highest expression. The effects of phosphatase treatment and purification of the mRNA were also investigated. Furthermore, EGFP expression was observed in yolk-sac salmon larvae following micro-injection. Conclusions: Our study provides an important basis for the development of efficient mRNA-based vaccines in the future.

## 1. Introduction

Farmed Atlantic salmon is an important food fish globally, and according to the Food and Agriculture Organization (FAO) of the United Nations (UN), the demand for animal-based food will increase in the years to come due to the predicted increase in the world’s population. Farmed fish, including farmed salmon, can make an important contribution to meet food needs [1]. In Norway, approximately 450 million salmon are vaccinated annually [2], and while most vaccines against bacterial diseases provide good protection, many of the currently available vaccines against viral diseases are not efficient enough. Full protection requires activation of cytotoxic T (Tc) cells, which can efficiently eliminate viruses during infection; however, most viral vaccines are made of inactivated viruses, and these will not induce Tc activation [3,4]. Another challenge is that some viruses are difficult to propagate due to a lack of an appropriate cell line, e.g., Piscine orthoreovirus (PRV), which causes heart and muscle inflammation (HSMI) in Atlantic salmon [5], and the non-virulent version (HRP0) of infectious salmon anemia virus (ISAV) [6]; for these diseases, vaccines have not been developed.

mRNA-based vaccines have emerged as a powerful alternative to conventional vaccines in humans, and mRNA vaccines have been developed against multiple infectious diseases [7,8,9]. They are considered safe as the mRNA is non-infectious and there is little risk of integration into the host genome because the mRNA does not enter the nucleus of transfected cells; it only needs to enter the cytosol following transfection to express proteins. The mRNA-based vaccine strategy is flexible, fast, and can be produced in a cell-free environment, as only the sequence of the antigen is needed, and thus it circumvents the challenge of virus propagation. In addition, it has been shown in both humans and mice that a single dose of mRNA vaccine elicited strong cellular and humoral immune responses [10,11,12]. Recently, two studies have shown enhanced green fluorescent protein (EGFP) expression from in vitro-transcribed (IVT) mRNA in fish cell lines and fish. Dahl et al. [13] showed EGFP expression in the Chum salmon heart cell line (CHH-1) cells and in salmon, and Ayad et al. [14] showed EGFP expression in Epithelioma papulosum cyprini (EPC) cells, Chinook salmon embryo (CHSE)-214 cells, and zebrafish. Ayad and coworkers also showed that an mRNA-based vaccine can protect fish against rhabdovirus infection.

A mature mRNA consists of a 5′ cap, 5′ untranslated region (UTR), an open reading frame (ORF), 3′ UTR, and a poly(A) tail. The ORF encodes the protein of interest, while the UTRs are important for translational regulation and mRNA stability. The 5′ cap is essential for initiation of protein synthesis as well as protection against exonucleases [15]. The naturally occurring 5′ cap structure in eukaryotic mature mRNAs is a N7-methylated guanosine (m^7^G) linked to the first nucleotide of the RNA via a 5′ to 5′-triphosphate bridge (m^7^GpppNp). A range of different 5′ cap structures is possible, such as cap 0, cap 1, and cap 2 (varying degrees of methylation). Addition of the 5′ cap can be done co-transcriptionally during in vitro transcription (IVT) using cap analogs or in a separate step after IVT using capping enzymes [16]. Two commonly used 5′ cap analogs in mRNA vaccines are the anti-reverse cap analog (ARCA) (m^7^(3′-O-methyl)-GpppG) and Clean Cap [16].

The 5′ and 3′ UTRs are important for regulating translation and maintaining mRNA stability [16]. 5′ UTRs in mRNA vaccines for humans are human alpha-globin, which is found in the BNT162b2/BioTech COVID-19 vaccine [17,18], human hydroxysteroid 17-beta-dehydrogenase 4 [16,19], and human dynein axonemal heavy chain 2 [16]. It has also been reported that optimization of the Kozak sequence can improve mRNA translation [20]. Regarding 3′ UTRs, hemoglobin subunit alpha is commonly used [16], but a study of cellular library and cell culture screening showed that a dual 3′ UTR of mitochondrially encoded 12S rRNA (MT-RNR1) and amino-terminal enhancer of split mRNA (AES) was superior to beta-globin 3′ UTR [21]. The dual MT-RNR1/AES 3′ UTR is included in the BNT162b2/BioTech vaccine. The IVT mRNAs encoding EGFP reported from fish contained 5′ and 3′ UTRs from hemagglutinin esterase [13].

The open reading frame encodes the protein of interest. Kariko and coworkers found that naturally occurring mRNA contains modified nucleosides, and that the modifications suppress recognition by Toll-like receptors (TLRs), allowing the immune system to distinguish between self and non-self mRNA. m6A in mRNA suppressed the activation of TLR3, while m6A, m5U, m5C, and Ψ prevented the stimulation of TLR7 and TLR8 [22,23]. Furthermore, they showed that the incorporation of Ψ into mRNA increased the longevity of the mRNA and the translation efficiency [24]. The incorporation of m1Ψ has been found to cause a greater reduction in the immunogenicity of the mRNA compared to Ψ [25] and is included in the human mRNA vaccines [26,27], as well as in mRNA in the study by Dahl et al. [13]. The mRNA may have an intrinsic adjuvant effect activating antigen-presenting cells (APCs), but if the activation is too strong, the mRNA may be degraded and/or the translation will be inhibited, leading to reduced protein expression and poor vaccine responses [28]. Teleost fish have many of the same TLRs as humans and mice [29], and most of the TLRs have the same ligand specificity, such as TLR3, TLR7, and TLR8, which recognize double-stranded RNA (dsRNA) and single-stranded RNA (ssRNA). Interestingly, the gene copy number of many TLRs varies among teleost species, e.g., Atlantic salmon has four TLR8s [30], while other species, such as lumpfish and carp, have only one [31,32]. Another TLR, specific for teleosts, is TLR22 [33,34]. These receptors recognize dsRNA, but they seem to have a broad ligand specificity, as it is also upregulated upon exposure to bacteria [29,35,36].

The poly(A) tail consists of approximately 70 nucleotides in yeast and 200 nucleotides in mammals [9,37]. In nature, the poly(A) tail is involved in the export of mRNA from the nucleus to the cytoplasm, stability of the mRNA, and translation into protein. The poly(A) tail is bound by poly(A) binding proteins (PABPs), which cover around 30 nucleotides each. Together with the m^7^G cap, the poly(A) tail recruits ribosomes and initiates translation. The m^7^G cap is recognized and bound by eukaryotic translation initiation factor 4E (eIF4E), which interacts with eIF4G. eIF4G then binds to PABPs, which brings the 5′ and 3′ ends together, forming a “closed loop” that initiates translation [37].

The aim of the current study was to optimize the mRNA technology for salmon. We constructed an mRNA tailored to salmon and evaluated the effect of modified nucleotides and the length of the poly(A) tail on protein expression. Moreover, we optimized the IVT protocol, using EGFP as a reporter, and verified the expression of EGFP in vitro and vivo using fluorescent microscopy and flow cytometry.

## 2. Materials and Methods

### 2.1. Templates for IVT

The plasmid used as a template for IVT was ordered from GenArt (Thermo Fisher Scientific). It was custom-made and contained a 5′ UTR from Atlantic salmon, and the EGFP and 3′ UTR sequences from the pEGFP-N1 plasmid. The plasmid was linearized with AflII (New England Biolabs, Ipswich, MA, USA) and purified with a PCR purification kit (Sigma-Aldrich, St. Louis, MO, USA). To make IVT-templates of known lengths of the poly(A) tail (40 As and 100 As), PCR was performed using the primers listed in Table 1 and Phusion DNA polymerase (Thermo Fisher Scientific, Vilnius, Lithuania). PCR products were purified using a PCR purification kit prior to IVT, and the DNA concentration was measured with a NanoDrop ND-1000 spectrophotometer (Nanodrop Technologies, Wilmington, DE, USA).

### 2.2. IVT

Linearized plasmid (0.5–1 μg) and PCR products (0.2 μg) were used as templates in the IVT. mRNAs encoding EGFP were synthesized using the mMESSAGE mMACHINE T7 Ultra Kit (InvitrogenVilnius, Vilnius, Lithuania). To make IVT mRNA without modified nucleosides, the kit was used according to the manufacturer’s instructions with a pre-made mix of nucleotides and ARCA cap. However, to make mRNA with modified nucleosides or without 5′ cap, ARCA cap (Invitrogen, Vilnius, Lithuania) and nucleotides (Thermo Scientific, Vilnius, Lithuania) were added separately, or ARCA was omitted in the reaction when making the mRNA without a 5′ cap. For example, to make mRNA with m6A, ATP was replaced with N6-methyladenosine-5′-triphosphate (TriLink Biotechnologies, San Diego, CA, USA). The other modified nucleotides were 5-methylcytidine-5′-triphosphate, 2-thiouridine-5′-triphosphate, 5-methyluridine-5′-triphosphate, pseudouridine-5′-triphosphate, and N1-methylpseudouridine-5′-triphosphate (all from TriLink). Other variables were phosphatase treatment of mRNA to remove uncapped 5′triphosphates, which may be recognized by pattern recognition receptors (PRRs) and initiate an immune response, and omit the purification step. The poly(A) tailing step was omitted to make mRNA without a poly(A) tail. PCR products were used as templates to make EGFP mRNA with 40-adenosine and 100-adenosine poly(A) tails, while the kit was used to make a poly(A) tail > 100 adenosines.

The mRNA was purified using the MEGAclear Kit (Invitrogen, Vilnius, Lithuania), according to the manufacturer’s instructions. The elution was performed twice. The eluted mRNA was stored at −80 °C or subjected to phosphatase treatment. For phosphatase treatment, the purified mRNA (~100 μL) was mixed with 1× Antarctic phosphatase buffer and Antarctic phosphatase (0.1 U/μL), and then incubated at 37 °C for 1 h. Thereafter, the sample was purified with the MEGAclear^TM^ Kit and stored at −80 °C.

### 2.3. Quantification and Quality Assessment of IVTmRNA

The concentration and purity of the RNA were measured using a NanoDrop ND-1000 spectrophotometer (Nanodrop Technologies) and/or Bioanalyzer (Agilent, Waldbronn, Germany). Bioanalyzer or 1% denaturing agarose gel electrophoresis using 1× MOPS buffer (Biosciences, St. Louis, MO, USA) was performed to assess the quality and size of the mRNA. Millennium RNA markers (Invitrogen, Vilnius, Lithuania) were used as markers. The denaturing gel contained 6.7% formaldehyde and GelRed (Biotium, Fremont, CA, USA).

### 2.4. In Vitro Culture of Salmonid Cell Line, Transfection, and Fluorescence Microscopy

CHSE-214 cells were cultured in 75 cm^2^ culture flasks at 20 °C without CO_2_ in Eagle's Minimum Essential Medium (EMEM) (Sigma, St. Louis, MO, USA) supplemented with 10% FBS (Gibco, Auckland, New Zealand), 0.1 mM non-essential amino acids (NEAAs) (Sigma, St. Louis, USA), 4 mM L-glutamine (Sigma-Aldrich, São Paulo, Brazil), and 50 μg/mL gentamicin (Grand Island, Grand Island, NY, USA). The cells were grown to approximately 90% confluency in culture medium before they were split or seeded into 24-well plates for transfection. The cells were seeded into wells 1 day prior to transfection and had a confluency of approximately 70% at the time of transfection. The culturing medium was replaced with medium without antibiotics before transfection. mRNA (0.5 μg) was mixed with Lipofectamine 3000 transfection reagent (Lipo3000) (Invitrogen, Carlsbad, CA, USA) or Lipofect MessengerMAX (LipoMM) (Invitrogen, Carlsbad, CA, USA), according to the manufacturer’s instructions, except that EMEM replaced Opti-MEM and the total volume per reaction was adjusted to 50 μL. The transfection solutions were incubated at room temperature for 20 min before they were added dropwise onto the cells. The cells were incubated at 20 °C for 1 to 10 days, and fresh medium was added after 5 days. To make samples for Western blot, CHSE cells were seeded into 6-well plates and transfected with 2 μg of mRNA mixed with Lipo3000. EGFP expression was monitored using Eclipse Ts2-FL microscopy (Nikon corporation, Tokyo, Japan).

### 2.5. Flow Cytometry to Measure EGFP Expression

CHSE-214 cells transfected with mRNAs (described in Section 2.2) encoding EGFP were harvested for flow cytometry, with five parallels per mRNA. The cells were washed once with PBS and loosened from the wells with trypsin. PBS + E (PBS with 1% BSA and 25 mM EDTA) was added to the wells, and the resuspended cells were transferred to 5 mL polystyrene tubes. The EGFP expression was measured with a BD FACScalibur^TM^ Flow cytometer (BD Biosciences, San Jose, CA, USA), and the following settings were used: FSC E00 V and 1.00 Amp Gain (linear), SSC 350 V and 1.00 Amp Gain (linear), and FL1 410–450 V and 1.00 Amp Gain (logarithmic). Gating the cells was performed to exclude dead cells and debris from subsequent analyses. Cells transfected with transfection reagents only were used as negative controls, and 0.2% of the negative controls were the limit to define the EGFP-positive cells. 10,000 cells were counted. The data was analyzed using FCS expression 7.30.0018 (De Novo Software).

### 2.6. Immunoblotting to Verify Expression of EGFP

Duplicate samples of CHSE-214 cells transfected with mRNAs encoding EGFP/Lipo3000 or transfection reagent only were assessed using 12% sodium dodecyl sulfate polyacrylamide gel electrophoresis (SDS-PAGE) and a Mini Protein Tetra cell (Bio-Rad Laboratories, Hercules, CA, USA). From the same gel, half of the gel (with one set of samples) was stained using Coomassie brilliant blue and the other half was subject to electroblotting and transferred to nitro-cellulose membranes (Bio-Rad) using a Bio-Rad mini transblot cell, and immunodetection was performed with rabbit-anti EGFP antibody (Chromotek, Planegg, Germany) diluted 1:1000. Secondary antibody, goat anti-rabbit conjugated with horseradish peroxidase (HRP) (Dako, Agilent Technologies, Glostrup, Denmark), was diluted 1:2000, developed using Clarity Western ECL Substrate (Bio-Rad), and pictures were taken with a GelDoc XR + Gel documentation system (BioRad). The membrane was stripped for antibodies, and then stained with anti-beta actin monoclonal antibody (Invitrogen, Rockford, IL, USA) diluted 1:5000 and anti-mouse secondary antibody conjugated with HRP (Dako, Agilent Technologies, Glostrup, Denmark) as described above.

### 2.7. RNA Isolation and qPCR of IVT mRNAs to Measure the Level of mRNA over Time

CHSE-214 cells were seeded into 24-well plates and transfected with EGFP-encoding mRNA with various lengths of the poly(A) tail as described above (Section 2.4). At given time points (1, 3, 6, and 10 dpt), the cell medium was removed, lysis buffer was added to the cells according to the manufacturer’s instructions, and the lysates were stored at −80 °C until RNA isolation. Total RNA was isolated using E.Z.N.A. Total RNA kit I (Omega, GA, USA), according to the manufacturer’s instructions, followed by DNase treatment and cDNA synthesis as described by Eggestøl et al. [38]. The quantity and quality of isolated RNA were measured/evaluated using a NanoDrop instrument (ND-1000 spectrophotometer, Nanodrop Technologies) and agarose gel, respectively. qPCR to monitor levels of IVT mRNA was measured as described by Eggestøl et al. [38]. Briefly, the PCR reactions (in total 10 µL) contained 5 µL 2× SYBR mix, 0.4 µL of each of the forward and reverse primers (10 µM), and 0.2 µL H_2_O. The forward primer (EGFP-Forw: 5′-TCTTCAAGGACGACGGCAAC-3′) and reverse primer (EGFP-R2: 5′-TCGATGCCCTTCAGCTCGAT-3′) were obtained from Sigma. Primers for the reference gene encoding ribosomal protein S20 (RPS20), also obtained from Sigma, have been described previously [39]. In the qPCR, three technical parallels were performed for each sample, and two negative controls were included for each triplicate mix: a cDNA reaction without reverse transcriptase (-RT) and a sample with water instead of cDNA (a non-template control, NTC). The qPCR was performed using a C1000 Touch Thermal Cycler with a CFX Real-Time System (BioRad) using the following program: 94 °C for 5 min, followed by 40 cycles of 15 c at 94 °C and 1 min at 60 °C. The presence of mRNAs encoding EGFP was normalized against RPS20 as described in Eggestøl et al. [38].

### 2.8. Injection of Salmon Larvae

Newly hatched yolk-sac salmon larvae were a kind gift from the Industrial and Aquatic Laboratory (ILAB) in Bergen, Norway. They were sedated in Finquel (MS-222) 25 mg/L, for approximately 3 min and injected with an mRNA/Lipo3000 mix using FemtoJet4i (Eppendorf, Hamburg, Germany) and glass capillary needles GDC-1 (Narishige Scientific instrument lab, Setagaya, Japan). The needles were made using PC-100 Puller (Narishige) in two steps, at 80 and 55 °C. Approximately 2 µL of mRNA/transfection reagent mix or transfection reagent only was added to the needles prior to injection. The larvae were recovered in seawater and thereafter kept in seawater at 15 °C for 3–7 days. Six larvae were injected with the mRNA/Lipo3000 mix and six larvae were injected with Lipo3000 only. The larvae were observed daily. Three larvae from each group were euthanized at days 3 and 7 post-injection. EGFP expression in the larvae was observed using a Leica DM 6000B microscope (Leica Microsystems, Wetzlar, Germany) with an ebx75mc-L90 lamp (Leistungselektronik Jena GmbH, Jena, Germany). Pictures were taken using a digital camera, Leica 350 FX (Leica Microsystems, Wetzlar, Germany). Ethical approval is not needed for experiments with yolk-sac larvae.

### 2.9. Statistics

Statistical analysis was performed with GraphPad Prism v10.4.2. One-way ANOVA with post hoc Tukey test was performed to determine statistical difference between groups. The results were considered statistically significant at *p* ≤ 0.05. Statistically significant groups are denoted with single letters, while non-significant groups share letters.

## 3. Results

### 3.1. Quantity and Quality Assessment of IVT mRNA

**Figure 1:**
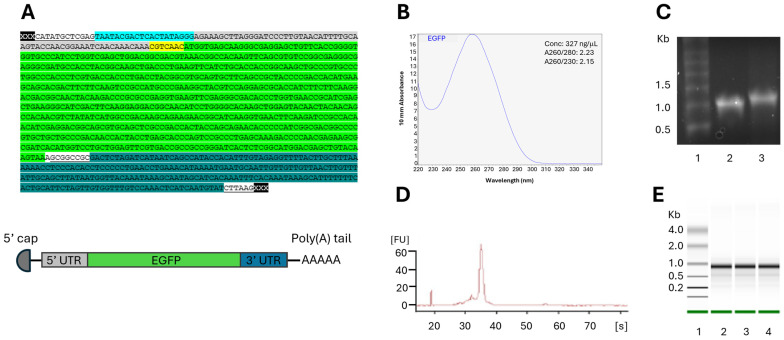
Quantification and quality assessment of IVT mRNA. (**A**) IVT template encoding EGFP. The different colored components are as follows: pMA vector, T7 promoter, 5′ UTR, Kozak sequence, EGFP, and 3′ UTR. The uncolored, underlined sequences are restriction enzyme sites. Below the sequence is a schematic overview of the mRNA. (**B**) NanoDrop measurement of purified IVT mRNA; (**C**) 1% denaturing agarose gel. Lane 1: Millennium RNA marker; Lane 2: IVT mRNA without a poly(A) tail; Lane 3: IVT mRNA with a poly(A) tail added by reagents from the kit; (**D**) electropherogram (Bioanalyzer); and (**E**) electrophoretic separation in Bioanalyzer of three parallel samples of IVT mRNA.

The template for the IVT mRNA contained a T7 promoter, 5′ UTR, a kozak sequence, EGFP sequence, and a 3′ UTR (Figure 1A). The quantity of the IVT mRNAs encoding EGFP was measured using a NanoDrop instrument. An outcome of a successful IVT typically yielded 300–450 ng/uL mRNA with A260/280 ~ 2.0 and A260/230 ~ 2.0 (Figure 1B). The size and quality of the mRNA were assessed with denaturing agarose gel (Figure 1C) and/or Bioanalyzer (Figure 1D,E). A clear band was seen for the mRNA (Figure 1C), and a size difference was observed between the mRNA with and without a poly(A) tail, showing a successful addition of the tail (Figure 1B). The poly(A)tail, added using the reagents provided by the IVT kit, was estimated to be approximately 0.15 kb, and this mRNA is called “poly(A) > 100”.

### 3.2. IVT mRNA Highly Expresses EGFP in the Salmonid Cell Line CHSE-214 and in Yolk-Sac Larvae

**Figure 2:**
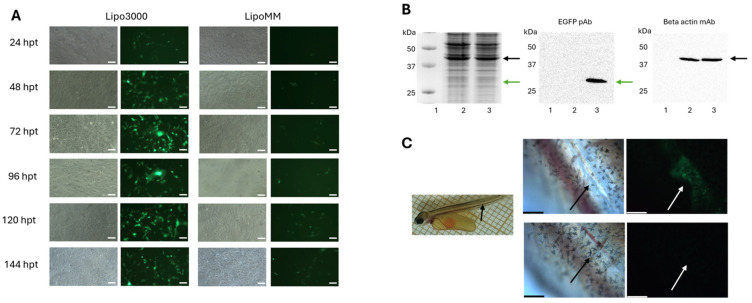
EGFP expression in CHSE-214 cells and in yolk-sac larvae. (**A**) Bright-field and fluorescence microscopy pictures were taken with 4× objective every 24 h up to 144 h. CHSE-214 cells in 24-well plates were transfected with 0.5 μg of mRNA with either Lipo3000 (left panels) or LipoMM (right panels) as a transfection reagent. Scale bar: 100 μm. (**B**) SDS–polyacrylamide gel (12%) (left panel) and Western blot of CHSE-214 cells transfected with mRNA. Lane 1: molecular weight standard (Precision Plus Protein^TM^ Kaleidoscope); Lane 2: control cells (transfection reagent only); and Lane 3: cells in 6-well plates transfected with 2 μg of mRNAs encoding EGFP. The green arrows in the middle and left panels indicate the expected size of EGFP (27 kDa), and the black arrows in the left and right panels indicate the expected size of beta-actin (42 kDa). (**C**) Left panel: injection point in yolk-sac larvae (black arrow). Middle panels: enlargement of the injection point (black arrow). Right panels: injection point (white arrow). Upper panels: larvae injected with mRNAs encoding EGFP. Green cells show cells expressing EGFP. Lower panels: larvae injected with transfection reagent only. Scale bar: 228 μm.

To assess the EGFP expression of mRNA tailored to salmon in a salmonid cell line, CHSE-214 cells were transfected. Two different commercially available transfection reagents were tested: Lipo3000 and LipoMM (Figure 2). EGFP expressions were seen in a fluorescent microscope, and strong EGFP expressions were observed 24–72 h post-transfection. The experiment was repeated several times, and a representative experiment is shown in Figure 2. Lipo3000 was undoubtedly a better transfection reagent than LipoMM and the one used in the following experiments. A Western blot with an anti-GFP antibody was performed to verify that the green fluorescence was from EGFP. The EGFP-encoding mRNA was also injected into yolk-sac larvae. Green fluorescence was seen in the area around the injection point, but not elsewhere in the larvae, confirming that the mRNA also expresses EGFP in vivo (Figure 2C). Larvae injected with the transfection reagent only did not show green fluorescence (Figure 2C).

### 3.3. A Poly(A) Tail > 100 bp Is Needed for Efficient Production of EGFP in CHSE-214

**Figure 3:**
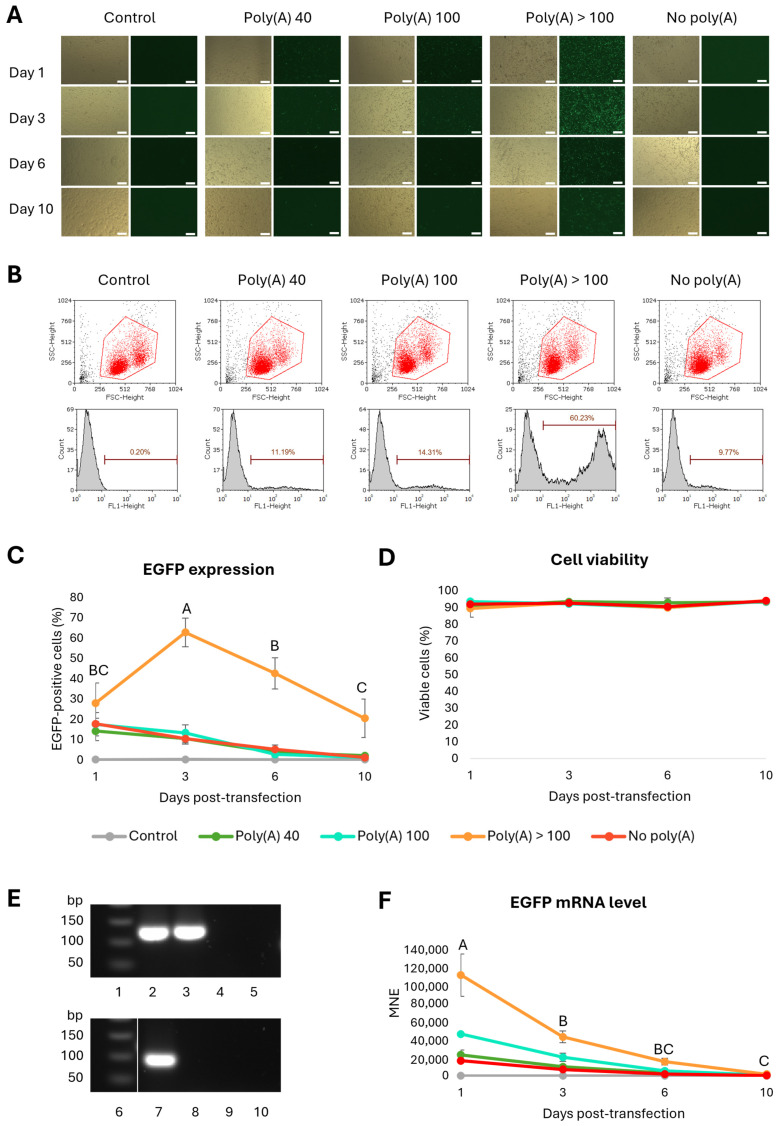
EGFP expression in CHSE-214 cells over time following transfection with mRNAs with different lengths of the poly(A) tail. (**A**) Fluorescence microscopy images taken with 4× objective 1, 3, 6, and 10 dpt. Scale bar: 200 μm. (**B**) Scatter plots and histograms of representative samples 3 days post-transfection with 0.5 μg mRNA. The red gates in the scatter plots define live cells (red dots), and the histograms show EGFP expression (FL1) for each of the mRNA constructs. The black dots to the lower left are cell debris. The markers represent EGFP-positive cells. (**C**) The diagram shows the average amount of EGFP-positive cells (%) after transfection with 0.5 μg mRNA lengths, analyzed 1, 3, 6, and 10 days post-transfection. *n* = 5. The error bars show the standard deviation (SD). (**D**) The diagram shows cell viability following transfection with the different mRNAs during the time course of the experiment. The error bars show the standard deviation. (**E**) The 2% agarose gel electrophoresis pictures of the qPCR products of RPS20 (upper panels) and EGFP (lower panels). Lanes 1 and 6: 50 bp DNA ladder; Lanes 2 and 7: gene expression in cells transfected with EGFP; Lanes 3 and 8: gene expression in cells that were exposed to transfection reagent only; Lanes 4 and 9: no RT-control; and Lanes 5 and 10: no template control. (**F**) Measurement of mRNA levels 1–10 days post-transfection. A one-way ANOVA was performed to compare EGFP expression of each of the different mRNAs over time. Single letters indicate that this measurement is statistically different from the others (*p* < 0.05). Shared letters indicate that the differences are not statistically significant. See Appendix A, Table A1, Table A2, Table A3 and Table A4, for statistical analyses.

**Figure A1:**
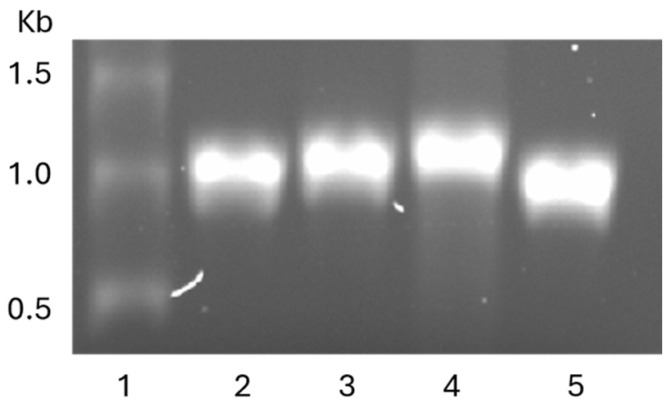
Denaturing agarose gel of mRNAs with different lengths of poly(A) tail. Lane 1: Millennium RNA marker; Lane 2: poly(A) 40, Lane 3: poly(A) 100, Lane 4: poly(A) > 100; and Lane 5: no poly(A).

To assess the effect of the poly(A) tail length on EGFP expression and mRNA stability, mRNAs with different lengths of the poly(A) tail were made. The sizes of the mRNAs were determined using denaturation agarose gel (Appendix A Figure A1). The IVT mRNA was transfected into CHSE-214 cells to assess the effect of tail length on EGFP expression and mRNA stability. An mRNA without a tail (termed no poly(A)) was included for comparison. On 1, 3, 6, and 10 days post-transfection (dpt), EGFP expression was monitored with fluorescence microscopy (Figure 3A) and measured by flow cytometry (Figure 3B,C). EGFP expression was only visible in the fluorescent microscope for the mRNA with the longest tail (poly(A) > 100), but when measured in flow cytometry, cells transfected with all mRNAs expressed EGFP (Figure 3A–C). The strongest expression was seen for Poly(A) > 100 3 days post-transfection (Figure 3B,C), which was significantly different from the other time points (Figure 3C) (Appendix A Table A1). It was also statistically higher than the other mRNAs and the control (Appendix A Table A2). Following day 3, the EGFP expression decreased over time for the poly(A) > 100 tail. For the other mRNAs, the highest expression was seen on day 1, after which the EGFP expression decreased. Analysis of cell viability showed between 89 and 95% cell viability in all the groups throughout the 10-day experiment (Figure 3D, Appendix A Table A3). To measure the level of mRNA, qPCR was performed using RPS20 as the reference gene. Both the RPS20 and EGFP assays were highly specific, giving one band of expected size (123 and 91 bp, respectively). There were no bands in any of the negative controls (Figure 3E). The mRNA levels of all the mRNAs declined throughout the experiment. The mRNA declined significantly between 1 and 3 days post-transfection (Figure 3F, Appendix A Table A4). Based on these results, the mRNA with a poly(A) tail > 100 adenosines was used in further experiments.

### 3.4. Effect of Modified Nucleosides, 5′ Cap, Purification, and Phosphatase Treatment on mRNA EGFP Expression

**Figure 4:**
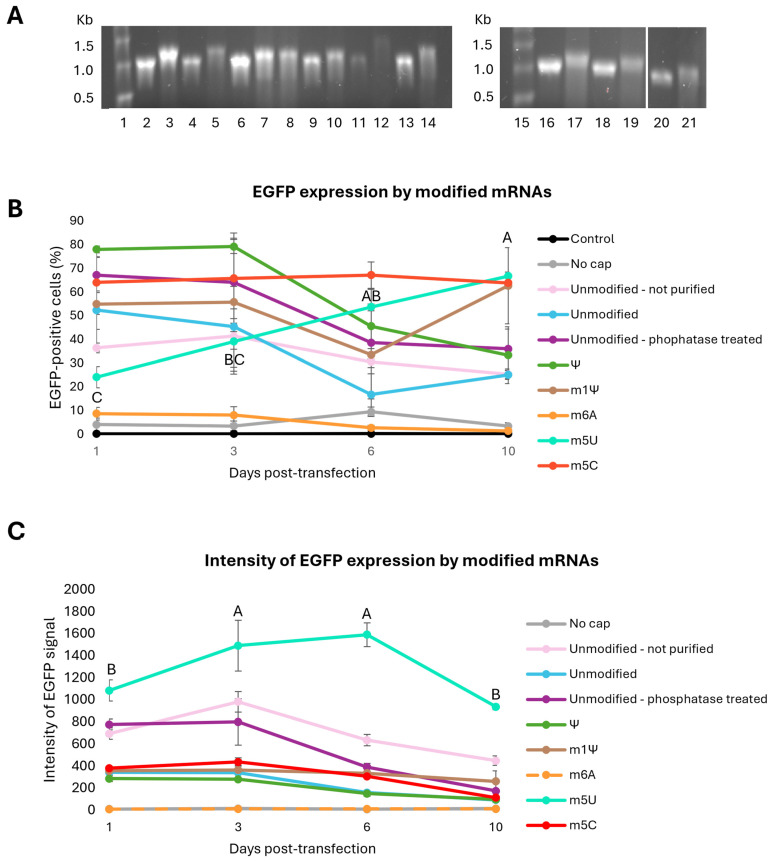
EGFP expression in CHSE-214 cells over time with mRNAs containing various modified nucleosides. (**A**) Denaturing agarose gel (1%) showing the different mRNAs prior to and after the addition of poly(A) tail. Lane 1: molecular weight standard (Millennium^TM^ RNA markers); Lanes 2–3: unmodified, not purified mRNA ± poly(A); Lanes 4–5: unmodified mRNA ± poly(A); Lanes 6–7: unmodified mRNA before phosphatase treatment ± poly(A); Lane 8: unmodified mRNA after phosphatase treatment + poly(A); Lanes 9–10: mRNA with Ψ ± poly(A); Lanes 11–12: mRNA with m6A ± poly(A); Lanes 13–14: mRNA with m5U ± poly(A); Lane 15: molecular weight standard (Millennium^TM^ RNA markers); Lanes 16–17: mRNA without 5′ cap ± poly(A); Lanes 18–19: mRNA with m1Ψ ± poly(A); and Lanes 20–21: mRNA with m5C ± poly(A). (**B**) The average percentage of EGFP-positive cells. (**C**) The intensity of the EGFP signal after transfection with 0.5 μg of mRNA containing modified nucleosides, unmodified mRNA with varying degrees of purification, or mRNA without a 5′ cap. Analyses were performed 1, 3, 6, and 10 dpt. *n* = 5. The error bars show standard deviation. A one-way ANOVA was performed to compare EGFP expression from different mRNAs for each time point. Single letters mean that this measurement is statistically different from the others (*p* < 0.05). Shared letters mean that the differences are not statistically significant. Significant differences are only shown for mRNAs with m5U. See Appendix A, Table A5, Table A6, Table A7 and Table A8, for all statistical analyses.

The next step was to investigate if modified nucleosides affected EGFP expression. Five mRNAs were made where one of the nucleotides was replaced with a modified one: Ψ, m1Ψ, m6A, m5U, or m5C. mRNA without modified nucleosides was also made (termed unmodified). Of the non-modified mRNAs, variants with the absence of purification and inclusion of phosphatase treatment were included: one mRNA was not purified, and one mRNA was phosphatase-treated and purified. Also, one mRNA was made without a 5′ cap (termed no cap). The quality of the mRNA was assessed with a denaturing agarose gel (Figure 4A), which showed that all mRNAs had bands around 1 kb before addition of the poly(A) tail, corresponding to its expected size of 1.025 Kb. The mRNAs were transfected into CHSE-214 cells, and the cells were harvested for flow cytometry 1, 3, 6, and 10 dpt. The highest percentage of EGFP-positive cells was found for the mRNA containing Ψ, which had 78 ± 2% positive cells at day 1 and 3, after which the EGFP expression decreased significantly (Figure 4B, Appendix A Table A5). Among the unmodified mRNAs, the phosphatase-treated mRNA showed the highest percentage of EGFP-positive cells (67 ± 7% on day 1). For this mRNA, along with the other unmodified mRNAs, the amount of EGFP-positive cells started decreasing after day 1 or 3. The mRNA containing m6A and the mRNA without a 5′ cap had low levels of EGFP-positive cells throughout the whole experiment and were not significantly different from the control. m1Ψ had 50–60% EGFP-positive cells for all days, except for day 6 where the amount was 34 ± 19%. mRNA containing m5U started at 24 ± 4% EGFP-positive cells on day 1, but increased to 67 ± 2% on day 10, while m5C-mRNA had a stable amount of EGFP-positive cells between 64 and 67% for each day of analysis (Appendix A Table A5). m1Ψ, m5U, and m5C had a significantly higher percentage of EGFP-positive cells on day 10 compared to the other mRNAs (Figure 4B, Appendix A Table A6).

To further compare EGFP expressions from the different mRNAs, the mean fluorescent intensity was analyzed for all mRNAs (Figure 4C). The strongest EGFP signal was seen for the mRNA containing m5U. The intensity at days 3 and 6 was significantly higher than days 1 and 10 (Appendix A, Table A7). The m5U mRNA had a significantly stronger signal compared to the other mRNAs for all time points (Figure 4C, Appendix A, Table A8).

## 4. Discussion

Viral diseases cause high mortality in the fish farming industry despite extensive vaccination [2,40]. Also, for some diseases, conventional vaccines (containing whole inactivated viruses) cannot be made due to a lack of cell lines to propagate the virus [41]. Therefore, there is a need for a new vaccine technology, and mRNA vaccines are a promising alternative. This vaccine technology requires only the sequence of a virus component, and circumvents problems associated with virus propagation in the lab, allowing more efficient and rapid vaccine production [16,42]. Also, the viral antigen can easily be changed. mRNA vaccines for humans protecting against viral diseases have successfully been developed and received full FDA approval [43]. The COVID-19 mRNA vaccines are the most known, but the development of mRNA vaccines against several diseases caused by, e.g., HIV and malaria, is ongoing, as well as vaccines for cancer [44]. As the essential immune molecules and biological processes are conserved between mammals and fish [45,46], mRNA vaccines should have the potential to elicit strong immune responses in fish as well. Clynav, a commercially available DNA vaccine against pancreas disease, gives good protection [47], and an mRNA vaccine protected rainbow trout against rhabdovirus infection [14]. This confirms the possibility of using nucleic acid vaccines for fish. However, to establish mRNA-based vaccines for a novel species, optimization of the different mRNA components is crucial, as it can highly affect expression of the protein of interest, and thus the vaccine efficacy.

In this study, the first steps were to verify that mRNA tailored to salmon expressed EGFP and to find a suitable transfection reagent. After transfection into CHSE-214 cells, EGFP expressions were seen in the cells transfected with mRNA and not in the control cells (transfection reagents only), confirming that the EGFP expression was caused by the mRNA and not by contaminated transfection reagents or autofluorescence. Unexpectedly, the Lipo3000 transfection reagent yielded higher EGFP expression than LipoMM, which is developed for the transfection of mRNA into a broad range of cells.

The impact of the poly(A) tail length on protein expression was investigated. Although long poly(A) tails have been associated with stabilization of the mRNA and translational initiation [48], more recent studies have found that highly expressed genes have shorter poly(A) tails. In the last larval stage of development in *Caenorhabditis elegans*, it was found that 90% of the mRNA molecules had poly(A) tails between 26 and 132 nucleotides, with the highest peak at 33–34 nucleotides [49]. Transcripts with longer poly(A) tails were detected for the highly expressed genes as well, suggesting that the nascent mRNAs go through a process that shortens the poly(A) tail, known as pruning [49]. In mammals, the length of the poly(A) tails immediately after polyadenylation is 150–250 nucleotides (nt), but in the cytoplasm, they undergo dynamic alterations characterized by shortening, and the steady-state length of the poly(A) tails is 80–100 nt [50]. In the current study, four mRNAs were made with different poly(A) tail lengths, 40, 100, and >100 adenosines, and an mRNA without a poly(A) tail. When transfected into CHSE-214 cells, EGFP was produced from all the mRNAs. The EGFP expression was, however, weak for all mRNAs except the one with the longest tail (>100 adenosines). This mRNA expressed significantly more EGFP than the others at all time points. The results show that a poly(A) tail longer than 100 adenosines is favorable for the protein expression and mRNA stability of IVT mRNA in salmonid cells. The results from other studies in fish, Dahl et al. [13] and Ayad et al. [14], are in accordance with this, as the mRNAs used in these studies had a poly(A) tail of 120 adenosines. In a study of human cells, where fibroblasts were reprogrammed to pluripotency using modified mRNA, a poly(A) tail of 120 was used [51].

Incorporation of modified nucleotides and purification of the mRNA have been shown to increase mRNA stability and protein expression, as well as make the mRNA less immunogenic [22,24,25,52]. For example, replacing uridine with m1Ψ reduces TLR7 binding due to steric exclusion in the ligand binding pocket, and thus bypasses innate immune responses and increases translation [27]. The human mRNA vaccines against COVID-19 contain m1Ψ [27]. The effect of incorporating modified nucleosides on EGFP expression in salmonid cells was assessed by transfecting mRNA with various modifications into CHSE-214 cells and measuring EGFP expression with flow cytometry. mRNA containing m6A yielded low expression, which was also seen for Kariko et al., where the transfection of mRNA containing m6A into 293 cells and bone marrow-derived murine dendritic cells caused low/no mRNA translation [24]. Kariko et al. also found that Ψ increased stability and caused a higher translational capacity than other nucleoside modifications, and this modified nucleoside was therefore suggested as a promising candidate for vaccination [24]. When transfecting EGFP-encoding mRNAs with modified nucleosides into CHSE-214 cells, the highest EGFP expression was found for Ψ up to 3 dpt, corresponding to the findings of Kariko et al. However, a decrease in the amount of EGFP-positive cells was seen after day 3, indicating that Ψ was not the best option for IVT mRNA in salmonid cells. A robust activation of the adaptive immune system will require a high and long expression of the antigen over time.

The most stable EGFP expression was seen for mRNA containing m5C. At 10 dpt, the m1Ψ-, m5U-, and m5C-containing mRNAs showed a significantly higher percentages of EGFP-positive cells compared to the other mRNAs. This is likely due to their ability to avoid recognition by TLRs, thereby increasing the translation efficiency [22,25]. m1Ψ had 50–60% EGFP-positive cells throughout the whole experiment, except for day 6. This modified nucleotide was used in the COVID-19 vaccines developed by Moderna and Pfizer/BioNTech [43] and can be a promising candidate for the mRNA vaccine against viral diseases for salmon. However, an even higher number of EGFP-positive cells was found for m5C, which had between 64 and 67% of EGFP-positive cells on each day of analysis. m5U did not have a high precentage of EGFP-positive cells at day 1, but ended up with the highest percentage of EGFP-positive cells on day 10. No significant differences were found between the EGFP expression of mRNA containing m1Ψ, m5C, or m5U on day 10. When looking at the intensity of the EGFP signal, m5U had a significantly stronger EGFP signal than all other mRNAs and should be considered when producing a vaccine. The mRNA without a 5′ cap had a low percentage of EGFP-positive cells throughout the whole experiment (less than 7%), highlighting the importance of a 5′ cap in mRNA translation. Furthermore, Linares-Fernández et al. found that the removal of dsRNA (purification) and uncapped mRNA (phosphatase treatment) before transfection into DCs caused a higher expression of EGFP [53]. This is likely due to impurities and uncapped mRNA, as this can initiate innate responses, which can lead to degradation of mRNA and/or inhibition of translation [12,28,53]. After transfection of CHSE-214 cells with EGFP-encoding mRNAs with various degrees of purification, a difference was seen in the amount of EGFP-positive cells in correlation to the level of purification, corresponding to the result of [53]. A significantly higher EGFP expression was found for the phosphatase-treated mRNA and the purified mRNA on day 1, compared to mRNA that was not purified.

## 5. Conclusions

In the current study, we constructed an mRNA tailored to salmon and evaluated the effect of the length of the poly(A) tail and modified nucleosides. We found that the poly(A) tail should be longer than 100 adenosines and that the mRNA containing m5U showed the highest protein expression. Our results also show that the transfection efficiency and EGFP expression are dependent on the presence of the 5′ cap and the degree of purification, and that it can be beneficial to include both purification and phosphatase treatment in the process of making mRNA. More investment in mRNA delivery and formulation of lipid nanoparticles tailored to salmon should be done, as well as exploration of how the modified nucleosides affect both innate and adaptive immune responses.

## Figures and Tables

**Table 1:** Oligonucleotides.

Primer Name	Sequence (5′-3′)
SS5UTRtail-F	GGAATTCCATATGCTCGAGTAATACG
SS5UTRtail40-R	TTTTTTTTTTTTTTTTTTTTTTTTTTTTTTTTTTTTTTTTCTTAAGATACATTGATGAGT
SS5UTRtail100-R	TTTTTTTTTTTTTTTTTTTTTTTTTTTTTTTTTTTTTTTTTTTTTTTTTTTTTTTTTTTTTTTT TTTTTTTTTTTTTTTTTTTTTTTTTTTTTTTTTTTTCTTAAGATACATTGATGAGT

## Data Availability

The original contributions presented in this study are included in the article. Further inquiries can be directed to the corresponding author.

## Appendix A

### Appendix A.1

**Table A1:** One-way ANOVA analysis of EGFP expression for each mRNA with different lengths of poly(A) tail over time *.

	Control	A40	A100	>A100	No p(A) Tail
Day 1	AB	A	A	BC	A
Day 3	A	A	A	A	B
Day 6	B	B	B	B	C
Day 10	AB	B	B	C	D

* Statistically significant groups are denoted with a single letter, while non-significant groups share letters.

**Table A2:** One-way ANOVA analysis of EGFP expression between mRNAs with different lengths of poly(A) tail at each time point *.

	Control	A40	A100	>A100	No p(A) Tail
Day 1	C	B	AB	A	AB
Day 3	C	B	B	A	B
Day 6	B	B	B	A	B
Day 10	B	B	B	A	B

* Statistically significant groups are denoted with a single letter, while non-significant groups share letters.

**Table A3:** One-way ANOVA analysis of cell viability for each mRNA with different lengths of poly(A) tail over time *.

	Control	A40	A100	>A100	No p(A) Tail
Day 1	A	B	A	A	B
Day 3	A	A	B	A	AB
Day 6	A	A	C	A	C
Day 10	A	A	A	A	A

* Statistically significant groups are denoted with a single letter, while non-significant groups share letters.

**Table A4:** One-way ANOVA of mean normalized expression for each mRNA with different lengths of poly(A) tail over time *.

	Control	A40	A100	>A100	No p(A) Tail
Day 1	A	A	A	A	A
Day 3	A	B	B	B	B
Day 6	A	B	C	BC	C
Day 10	A	B	C	C	C

* Statistically significant groups are denoted with a single letter, while non-significant groups share letters.

**Table A5:** One-way ANOVA analysis of EGFP expression for each modified mRNA over time *.

	Control	No Cap	Unmodified Not Purified	Unmodified	Unmodified PhosphaseTreated	Ψ	m1Ψ	m6A	m5U	m5C
Day 1	A	B	AB	A	A	A	A	A	C	A
Day 3	A	B	A	AB	AB	A	A	A	BC	A
Day 6	A	A	BC	C	BC	B	A	B	AB	A
Day 10	A	B	C	BC	C	B	A	B	A	A

* Statistically significant groups are denoted with a single letter, while non-significant groups share letters.

**Table A6:** One-way ANOVA analysis of EGFP expression between modified mRNAs at each time point *.

	Control	No Cap	Unmodified Not Purified	Unmodified	Unmodified Phosphatase Treated	Ψ	m1Ψ	m6A	m5U	m5C
Day 1	F	EF	CD	BC	AB	A	BC	EF	DE	AB
Day 3	D	D	BC	AC	AB	A	AB	CD	BC	AB
Day 6	E	E	CD	DE	BC	BC	CD	E	AB	A
Day 10	C	C	B	B	B	B	A	C	A	A

* Statistically significant groups are denoted with a single letter, while non-significant groups share letters.

**Table A7:** One-way ANOVA analysis of EGFP signal intensity for each modified mRNA over time *.

	No Cap	Unmodified Not Purified	Unmodified	Unmodified Phosphatase Treated	Ψ	m1Ψ	m6A	m5U	m5C
Day 1	A	B	A	A	A	A	B	B	B
Day 3	A	A	A	A	A	A	AB	A	A
Day 6	A	B	B	B	B	A	AB	A	C
Day 10	A	C	B	B	C	A	A	B	D

* Statistically significant groups are denoted with a single letter, while non-significant groups share letters.

**Table A8:** One-way ANOVA analysis of EGFP signal intensity between modified mRNAs at each time point *.

	No Cap	Unmodified Not Purified	Unmodified	Unmodified Phosphatase Treated	Ψ	m1Ψ	m6A	m5U	m5C
Day 1	D	B	C	B	C	C	D	A	C
Day 3	D	B	C	B	CD	C	D	A	C
Day 6	E	B	D	C	D	C	E	A	C
Day 10	E	B	DE	CD	DE	C	E	A	D

* Statistically significant groups are denoted with a single letter, while non-significant groups share letters.
